# Laminin 221 fragment is suitable for the differentiation of human induced pluripotent stem cells into brain microvascular endothelial-like cells with robust barrier integrity

**DOI:** 10.1186/s12987-020-00186-4

**Published:** 2020-03-30

**Authors:** Hiromasa Aoki, Misaki Yamashita, Tadahiro Hashita, Takahiro Iwao, Tamihide Matsunaga

**Affiliations:** Department of Clinical Pharmacy, Graduate School of Pharmaceutical Sciences, Nagoya City University, 3-1 Tanabe-dori, Mizuho-ku, Nagoya, 467-8603 Japan

**Keywords:** Human induced pluripotent stem cell, Brain microvascular endothelial cell, Laminin 221, Differentiation, Blood–brain barrier

## Abstract

**Background:**

In vitro blood–brain barrier (BBB) models using human induced pluripotent stem (iPS) cell-derived brain microvascular endothelial-like cells (iBMELCs) have been developed to predict the BBB permeability of drug candidates. For the differentiation of iBMELCs, Matrigel, which is a gelatinous protein mixture, is often used as a coating substrate. However, the components of Matrigel can vary among lots, as it is obtained from mouse sarcoma cells with the use of special technics and also contains various basement membranes. Therefore, fully defined substrates as substitutes for Matrigel are needed for a stable supply of iBMELCs with less variation among lots.

**Methods:**

iBMELCs were differentiated from human iPS cells on several matrices. The barrier integrity of iBMELCs was evaluated based on transendothelial electrical resistance (TEER) values and permeability of fluorescein isothiocyanate-dextran 4 kDa (FD4) and Lucifer yellow (LY). Characterization of iBMELCs was conducted by RT-qPCR and immunofluorescence analysis. Functions of efflux transporters were defined by intracellular accumulation of the substrates in the wells of multiwell plates.

**Results:**

iBMELCs differentiated on laminin 221 fragment (LN221F-iBMELCs) had higher TEER values and lower permeability of LY and FD4 as compared with iBMELCs differentiated on Matrigel (Matrigel-iBMELCs). Besides, the gene and protein expression levels of brain microvascular endothelial cells (BMEC)-related markers were similar between LN221F-iBMELCs and Matrigel-iBMELCs. Moreover, both Matrigel- and LN221F-iBMELCs had functions of P-glycoprotein and breast cancer resistance protein, which are essential efflux transporters for barrier functions of the BBB.

**Conclusion:**

The fully defined substrate LN221F presents as an optimal coating matrix for differentiation of iBMELCs. The LN221F-iBMELCs had more robust barrier function for a longer period than Matrigel-iBMELCs with characteristics of BMECs. This finding will contribute the establishment of an iBMELC supply system for pharmacokinetic and pathological models of the BBB.

## Background

Brain microvascular endothelial cells (BMECs), which are one of the constituents of the blood–brain barrier (BBB), regulate the transition of substances between the vasculature and brain parenchyma via strong intercellular adhesions and expression of multidrug efflux transporters [1]. In drug discovery, the robustness of the BBB often abrogates drug development for the treatment of central nervous system disorders because many candidates cannot reach the brain parenchyma. To predict drug permeability of the BBB, in vivo murine and monkey models are often used [2]. However, drug permeability of the BBB of these models differs from that of humans [3] and the use of in vivo models is both expensive and time-consuming. Therefore, it is difficult to predict the permeability of all drug candidates using in vivo models. Hence, it is necessary to establish models that can easily and precisely predict drug permeability of the BBB for pre-screening, especially for drug discovery.

Various in vitro BBB models, including immortalized human BMECs and rodent-derived BMECs, have been reported [4]. However, immortalized BMECs have weak barrier functions, and thus, are not suitable to predict drug permeability [5]. Besides, in case of using rodent-derived BMECs, interspecies differences between rodent and human BMECs are problematic. To address these problems, human induced pluripotent stem (iPS) cell-derived brain microvascular endothelial-like cells (iBMELCs) were recently developed [6, 7]. Transendothelial electrical resistance (TEER), which is an index of the robustness of tight junctions, of iBMELCs is comparable to that of the BBB in vivo [7]. Therefore, it is expected that drug permeability of the human BBB can be predicted with the use of iBMELCs.

It is necessary to establish a stable supply system of large numbers of iBMELCs as drug screening models. Matrigel, which contains various basement membrane components such as laminin, collagen, and entactin, is often used for the differentiation of iBMELCs [6–10]. However, the components of Matrigel can vary among various batches because it is extracted from mouse sarcoma cells using special techniques. In addition, the quality of the Matrigel influences the barrier integrity of iBMELCs [11]. Moreover, Matrigel coating requires complex manipulations because Matrigel solidifies with an increase in temperature. Therefore, it is difficult to ensure the reproducibility of differentiation with the use of Matrigel. Therefore, Matrigel is not suitable for reproducible production of large numbers of iBMELCs as a fully defined substrate should be used for differentiation. Here, we show that coating of defined substrate laminin 221 fragment (LN221F), which is a recombinant fragment with full integrin-binding activity, enhanced the barrier integrity of iBMELCs. Moreover, gene and protein expression levels of endothelial cell markers, efflux transporters, tight junction-related proteins, and other BMEC-related markers in iBMELCs differentiated on LN221F (LN221F-iBMELCs) were comparable to those of iBMELCs differentiated on Matrigel (Matrigel-iBMELCs). In addition, LN221F-iBMELCs had functions of efflux transporters, which are essential for the barrier functions of the BBB. Considering that coating a culture plate with LN221F requires only simple manipulation, the findings of this study are expected to contribute to the establishment of a stable supply system of BBB models with robust barrier functions for a prolonged period.

## Materials and methods

### Materials

The human iPS cell lines 610B1 and 648A1 derived from human umbilical cord blood and human peripheral blood, respectively, were purchased from RIKEN BioResource Center (Tsukuba, Japan). The immortalized human BMEC cell line hCMEC/D3 was purchased from Merck Millipore (Burlington, MA, USA). The primary human BMECs and Complete Classic Medium with Serum and CultureBoost™ were purchased from Cell Systems Corporation (Kirkland, WA, USA). The primary BMECs derived from a monkey (*Macaca irus*) and the culture medium for BMECs were purchased from PharmaCo-Cell Co., Ltd. (Nagasaki, Japan). Fibronectin (FBN), StemSure^®^ hPSC medium, l-glutamine, 1:1 mixture of Dulbecco’s modified Eagle’s medium and Ham’s nutrient mixture F-12 (DMEM/F12), hydrocortisone, l-ascorbic acid phosphate magnesium salt n-hydrate, and MEM nonessential amino acids were purchased from Wako Pure Chemical Industries, Ltd. (Osaka, Japan). Endothelial Cell Basal Medium 2 was purchased from Lonza (Basel, Switzerland). Porcine skin gelatin, 2-mercaptoethanol, fetal bovine serum, and 4-(2-hydroxyethyl)-1-piperazineethanesulfonic acid (HEPES) solution (1 M, pH 7.0–7.6) were purchased from Sigma-Aldrich Corporation (St. Louis, MO, USA). LN221F, laminin 411 fragment (LN411F), and laminin 511 fragment (LN511F) (imatrix 221, 411, and 511, respectively) were purchased from Nippi Incorporated (Tokyo, Japan). Gibco™ KnockOut™ Serum Replacement (KSR), Gibco™ Hank’s Balanced Salt Solution (HBSS) with Calcium and Magnesium, No Phenol Red, Human Endothelial-Serum-Free Medium (HE-SFM), Gibco™ Chemically Defined Lipid Concentrate, and Vitronectin-N (VTN-N) were purchased from Thermo Fisher Scientific (Waltham, MA, USA). Fibroblast growth factor-2 (FGF2) was purchased from PeproTech, Inc. (Rocky Hill, NJ, USA). Penicillin–streptomycin solution was purchased from Biological Industries USA, Inc. (Cromwell, CT, USA). An Agencourt RNAdvance Tissue Total RNA Purification Kit was purchased from Beckman Coulter, Inc. (Brea, CA, USA). ReverTra Ace^®^ qPCR RT Master Mix was purchased from Toyobo Co., Ltd. (Osaka, Japan). KAPA SYBR^®^ FAST qPCR Master Mix (2×) was purchased from Nippon Genetics Co., Ltd. (Tokyo, Japan). Corning^®^ Matrigel^®^ Growth Factor Reduced (GFR) Basement Membrane Matrix, Falcon^®^ Permeable Support for a 24-well plate with a 0.4-µm Transparent PET Membrane, and a Falcon^®^ 24-well TC-treated Cell Polystyrene Permeable Support Companion Plate and a lid were purchased from Corning Incorporated (Corning, NY, USA). Platelet-poor plasma-derived bovine serum (PDS) and 1,1′-Dioctadecyl-3,3,3′,3′-tetramethyl-indocarbocyanine perchlorate acetylated low-density lipoprotein (Dil-Ac-LDL) were purchased from Alfa Aesar (Ward Hill, MA, USA). All-*trans* retinoic acid (RA) was purchased from Tocris Bioscience (Bristol, UK). Accutase™ was purchased from Nacalai Tesque, Inc. (Kyoto, Japan). Cell Carrier-96 Black, Optically Clear Bottom microplates were purchased from PerkinElmer, Inc. (Waltham, MA, USA). Collagen type IV was purchased from Nitta Gelatin Inc. (Osaka, Japan). Total RNA from human primary BMECs (hBMECs) was purchased from ScienCell Research Laboratories, Inc. (Carlsbad, CA, USA).

### Coating before differentiation

Matrigel GFR was diluted (1:30) in iPS cell medium on ice. The diluted solution was transferred into the wells of a 6-well plate with a cold tip and incubated at 37 °C for 1 h. FBN, VTN-N, LN221F, LN411F, and LN511F, which were diluted (1 μg/cm^2^) with D-phosphate-buffered saline (D-PBS) (−), were transferred into the wells of six-well plates and incubated at 37 °C for 1 h.

### Cell culture

Human iPS cells were maintained in iPS cell medium (DMEM/F12 containing 20% KSR, 2 mM l-glutamine, 1 × MEM nonessential amino acids, and 0.1 mM 2-mercaptoethanol) supplemented with 5 ng/mL of FGF2. The human iPS cells were cultured on mitomycin C-treated mouse embryonic fibroblasts with daily medium changes. The primary human BMECs were cultured in Complete Classic Medium with Serum supplemented with CultureBoost. The cells were dissociated with TrypLE select and plated on dishes coated with Attachment Factor at a 1:3 split ratio. Before conducting the TEER measurement assay, the cells (passage 6) were seeded onto transwell culture inserts coated with Matrigel, FBN, VTN-N, LN221F, LN411F, or LN511F at a density of 5.0 × 10^4^ cells/well and cultured in HE-SFM-based medium (HE-SFM containing 1% PDS and 1 × penicillin–streptomycin solution) supplemented with 20 ng/mL of FGF2. The medium was changed every alternate day. For the TEER measurement assay of the primary monkey BMECs, the cryopreserved cells (passage 1) were thawed and seeded onto transwell culture inserts coated with Matrigel, FBN, VTN-N, LN221F, LN411F, or LN511F at a density of 2.0 × 10^4^ cells/well and cultured in the culture medium for BMECs. The medium was changed every alternate day. The hCMEC/D3 cells were cultured in hCMEC/D3 medium: Endothelial Cell Basal Medium 2 supplemented with 5% fetal bovine serum, 5 μg/mL l-ascorbic acid phosphate magnesium salt n-hydrate, 1% chemically defined lipid concentrate, 10 μM HEPES solution, 1 × penicillin–streptomycin solution, 1.4 μM hydrocortisone, and 1 ng/mL FGF2. The cells were dissociated with TrypLE select and plated at a 1:5 split ratio. Before the TEER measurement assay, the cells were seeded onto transwell culture inserts coated with Matrigel, FBN, VTN-N, LN221F, LN411F, or LN511F at a density of 5.0 × 10^4^ cells/well and cultured in HE-SFM-based medium supplemented with 20 ng/mL of FGF2. The medium was changed every alternate day.

### Differentiation of iPS cells into iBMELCs

iBMELCs were differentiated from human iPS cells using a modified protocol of a previous study [7]. Briefly, iPS cells were seeded into the wells of a 6-well plate coated with Matrigel GFR, FBN, VTN-N, LN221F, LN411F, or LN511F and cultured with StemSure hPSC medium supplemented with 35 ng/mL of FGF2 for 3 or 4 days. When cell counts were needed, we prepared human iPS cells in two culture dishes, one each for differentiation and cell count. On day 0, after reaching 60–70% confluence, the culture medium was replaced with DMEM/F12-based medium (iPS cell medium) and the cells were cultured for an additional 6 days. The medium was changed every day. On day 6, the medium was switched to HE-SFM-based medium supplemented with 10 μM RA and 20 ng/mL of FGF2. On day 8, the differentiated cells were washed with D-PBS (–) and dissociated with the use of Accutase cell detachment solution for 20 min at 37 °C. The cells were centrifuged at 100×*g*, resuspended, seeded on a transwell culture insert or into the wells of a multiwell plate coated with a mixture of FBN (100 μg/mL) and collagen type IV (400 μg/mL), LN221F (100 μg/mL), or a mixture of FBN, collagen type IV, and LN221F at a density of 3.0 × 10^5^ cells/well, and cultured in HE-SFM-based medium supplemented with 10 μM RA and 20 ng/mL of FGF2. On day 9, the medium was switched to HE-SFM-based medium without 10 μM RA and 20 ng/mL of FGF2.

### Cell viability assay

CCK-8 assay was conducted in accordance with the manufacturer’s instructions. The cells were incubated with CCK-8 solution for 60 min.

### TEER value measurement

TEER values were measured with the use of a Millicell^®^ ERS-2 V/Ωm (Merck Millipore) in accordance with the manufacturer’s instructions.

### Paracellular permeability assay

The iBMELCs were cultured on a transwell culture insert. On day 10, the medium was replaced with transport buffer (HBSS containing 10 mM HEPES solution) and the cells were cultured for 20 min at 37 °C. Transport buffer containing 1 mg/mL of FD4 or 300 μM LY was added to the apical side and transport buffer alone was added to the basolateral side. After 60 min of incubation at 37 °C, 100 μL of the solution was collected from the basolateral side. The volumes of transport buffer in the apical and basal sides were 300 and 800 μL, respectively. Fluorescent signals of FD4 or LY were measured with a Synergy HTX multimode plate reader and analyzed using Gen 5 data analysis software (BioTek Instruments, Inc., Winooski, VA, USA).

### RNA extraction and RT-qPCR

Total RNA was purified using an Agencourt RNAdvance Tissue Total RNA Purification Kit and reverse transcribed using ReverTra Ace qPCR RT Master Mix. Relative mRNA expression levels were measured using the KAPA SYBR^®^ FAST qPCR Kit with a LightCycler^®^ 96 System (F. Hoffmann-La Roche, Ltd., Basel, Switzerland) and normalized to those of hypoxanthine guanine phosphoribosyltransferase 1 (HPRT1). The RT-qPCR primers are listed in Table 1.

**Table 1:** RT-qPCR primer sequences

Genes	Forward primer sequence (5′ → 3′)	Reverse primer sequence (5′ → 3′)
*CDH5*	GATTTGGAACCAGATGCACA	ACTTGGCATTCTTGCGACTC
*MDR1*	CCCATCATTGCAATAGCAGG	TGTTCAAACTTCTGCTCCTGA
*BCRP*	AGATGGGTTTCCAAGCGTTCAT	CCAGTCCCAGTACGACTGTGACA
*GLUT1*	GAAGAGAGTCGGCAGATGATG	GGAGTAATAGAAGACAGCGTTGATG
*Occludin*	TCCAATGGCAAAGTGAATGA	GCAGGTGCTCTTTTTGAAGG
*ZO*-*1*	CGAGGGATAGAAGTGCAAGTAGA	TATTCTTCATTTTTCCGGGATTT
*LAT1*	AATGGGTCCCTGTTCACATC	CGTAGAGCAGCGTCATCACA
*HPRT1*	CTTTGCTTTCCTTGGTCAGG	TCAAGGGCATATCCTACAACA
*PECAM1*	AGTCGGACAGTGGGACGTAT	ATGACCTCAAACTGGGCATC
*vWF*	CGGCTTGCACCATTCAGCTA	TGCAGAAGTGAGTATCACAGCCATC
*KDR*	CTGCAAATTTGGAAACCTGTC	GAGCTCTGGCTACTGGTGATG
*TBXT*	ACCCAGTTCATAGCGGTGAC	CAATTGTCATGGGATTGCAG
*KDR*	CTGCAAATTTGGAAACCTGTC	GAGCTCTGGCTACTGGTGATG
*OCT*-*4*	AGCGAACCAGTATCGAGAAC	TTACAGAACCACACTCGGAC
*NANOG*	CTGCAGAGAAGAGTGTCGCA	ACCAGGTCTTCACCTGTTTG

### Immunofluorescence analysis

For staining of cadherin 5 (CDH5, also known as VE-cadherin), claudin 5, P-glycoprotein (P-gp), and breast cancer resistance protein (BCRP), cells in the wells of Cell carrier-96 microplates were washed thrice with D-PBS (−) containing 0.1% bovine serum albumin (BSA), fixed with 4% paraformaldehyde for 15 min, washed thrice with D-PBS (–) containing 0.1% BSA, and permeabilized with D-PBS (−) containing 0.1% Triton X100 for 5 min. After washing thrice with D-PBS (−) containing 0.1% BSA, the cells were incubated overnight at 4 °C with a primary antibody diluted in D-PBS (−) containing 0.1% BSA. Afterward, the cells were washed thrice with D-PBS (−) containing 0.1% BSA and incubated with a secondary antibody for 60 min at room temperature. After washing thrice with D-PBS (−) containing 0.1% BSA, the cells were incubated with 1 µg/mL of 4′,6-diamidino-2-phenylindole (DAPI) for 5 min. The cells were then treated with 4% paraformaldehyde for 5 min and washed thrice times with D-PBS (−).

For staining of zonula occludens-1 (ZO-1) and occludin, cells in the wells of Cell carrier-96 microplates were fixed with 4% paraformaldehyde for 15 min, washed thrice with D-PBS (−) containing 10 mM glycine, permeabilized in D-PBS (−) containing 0.1% Triton X100 for 25 min, and stored in D-PBS (−) containing 0.1% NaN_3_ at 4 °C. The stored cells were blocked with 5% donkey serum for 20 min, incubated with a primary antibody for 120 min, and then incubated with a secondary antibody and 1 µg/mL DAPI for 60 min.

The stained samples were visualized using an Operetta High-Content Imaging System (PerkinElmer, Inc., Waltham, MA, USA). The antibodies used in this study are shown in Table 2. The fluorescence intensities were also calculated using Harmony^®^ high-content analysis software (PerkinElmer, Inc., Waltham, MA, USA). The mean fluorescence intensities were calculated by dividing the fluorescence intensity by the total cell number.

**Table 2:** Antibodies for immunofluorescence analysis

Targets	Manufacturer	Catalog number	Species	Dilution
CDH5	Santa Cruz	sc-9989	Mouse	1:25
P-gp	Abcam	ab10333	Mouse	1:25
BCRP	Abcam	ab3380	Mouse	1:50
Occludin	Fisher Scientific	71-1500	Rabbit	1:50
ZO-1	Fisher Scientific	33-9100	Mouse	1:100
Claudin-5	Fisher Scientific	35-2500	Mouse	1:50
Anti-Rabbit (Alexa Flour 488)	Fisher Scientific	A-21206	Donkey	1:200
Anti-Mouse (Alexa Fluor 568)	Fisher Scientific	A-11004	Goat	1:200

### Dil-Ac-LDL uptake assay

Differentiated cells were first incubated with 10 μg/mL Dil-Ac-LDL for 5 h, then incubated with 10 µg/mL Hoechst 33342 at room temperature for 10 min, and washed four times with medium. The samples were visualized using an Operetta High-Content Imaging System. The fluorescence intensities of Dil were also calculated using Harmony^®^ high-content analysis software. The mean fluorescence intensities were calculated by dividing the intensity of Dil by the total cell number.

### Functional analysis of efflux transporters

The culture medium was removed and the cells in the wells of a 96-well plate were pre-incubated with transport buffer (HBSS containing 10 mM HEPES buffer) at 37 °C for 15 min. Intracellular accumulation was initiated by the replacement of transport buffer containing 10 μM rhodamine 123 or 10 μM Hoechst 33342 at 37 °C in the presence or absence of 10 μM cyclosporine A (CsA) or 20 μM Ko 143, which are inhibitors of P-gp and BCRP, respectively. After 60 min, the cells were washed thrice with D-PBS (−) and lysed using D-PBS (−) containing 5% Triton X-100. The fluorescence intensities were measured using a Synergy HTX multimode plate reader and analyzed with Gen 5 data analysis software.

### Western blot analysis

The differentiated cells on day 10 were lysed in 1× sodium dodecyl sulfate–polyacrylamide gel electrophoresis (SDS-PAGE) sample buffer. The protein samples were separated by SDS-PAGE, transferred to polyvinylidene fluoride membranes, which were blocked with 4% Block-Ace solution, washed with tris-buffered saline containing 0.1% Tween 20 (TBS-T), incubated with primary antibodies (Table 3) at room temperature for 2 h, and then incubated with secondary antibodies at room temperature for 30 min (Table 3). After washing with TBS-T, Protein bands were detected using an Amersham Imager 600 system (GE Healthcare Life Sciences, Chicago, IL, USA). The protein bands were quantified using Amersham Imager 600 analysis software. Background signals were removed with the Rolling Ball algorithm (Amersham Imager 600 analysis software).

**Table 3:** Antibodies for Western blotting analysis

Targets	Manufacturer	Catalog number	Species	Dilution
Occludin	Fisher Scientific	71-1500	Rabbit	1:500
ZO-1	Fisher Scientific	33-9100	Mouse	1:500
Claudin-5	Fisher Scientific	35-2500	Mouse	1:500
GAPDH (HRP)	Wako	35-2500	Mouse	1:1000
Anti-Mouse (HRP)	Abcam	ab6789	Goat	1:5000
Anti-Rabbit (HRP)	Abcam	ab6721	Goat	1:5000

### Statistical analysis

For all experiments, ‘‘n’’ represents the number of biological replicates (three or six wells were analyzed). Each experiment was repeated at least twice. Data are presented as the mean ± standard deviation (SD). The two-tailed Student’s *t*-test was used for comparisons between two groups. Two-way repeated measures analysis of variance was used for comparisons of TEER values measured after a prolonged period. One-way analysis of variance followed by Tukey’s honestly significant difference test or the Games–Howell test was used for comparisons of all six groups. Levene’s test was used to assess the equality of variances. All statistical analyses were performed using IBM SPSS Statistics for Windows, version 25.0 (IBM Corporation, Armonk, NY, USA). A probability (*p*) value of < 0.05 was considered statistically significant.

## Results

### LN221F coating enhanced the barrier integrity of iBMELCs

**Fig. 1:**
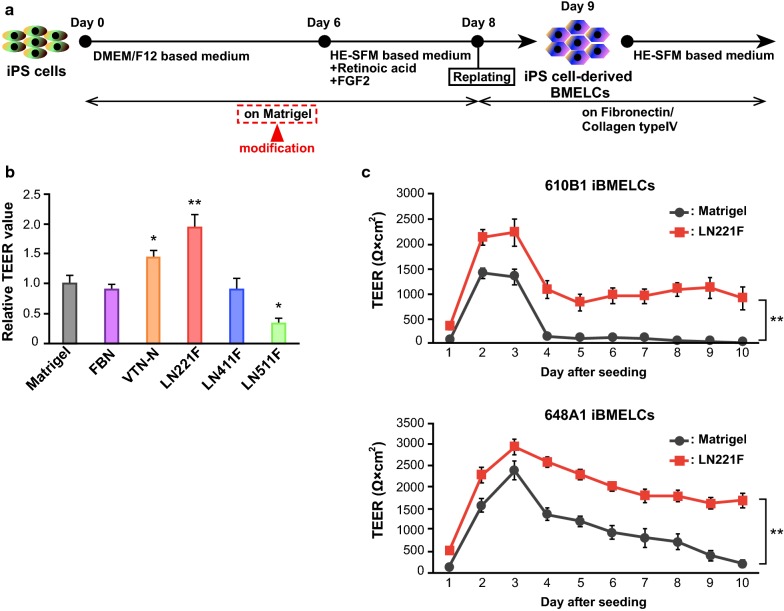
Evaluation of TEER values in LN221F- and Matrigel-iBMELCs. **a** Scheme for the differentiation of iBMELCs. **b** Relative TEER values of 610B1-derived iBMELCs on day 10 on Matrigel, FBN, VTN-N, LN221F, LN411F, or LN511F. The relative TEER value of iBMELCs on Matrigel was defined as 1. Data are presented as the mean ± SD (*n* = 3; *p < 0.05, **p < 0.01; Tukey’s honestly significant difference test, Matrigel group vs. others). **c** Measurement of TEER values of LN221F- and Matrigel-iBMELCs derived from 610B1 and 648A1 over a long period (from day 9 to 18). Data are presented as the mean ± SD (*n *= 6; **p < 0.01; two-way repeated measures analysis of variance)

**Fig. 2:**
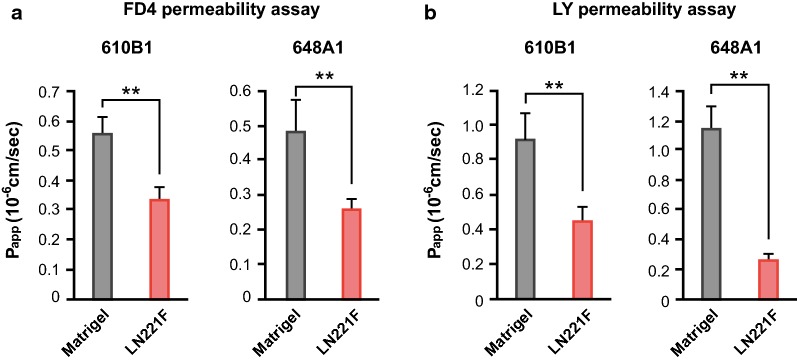
Analyses of paracellular permeability of LN221F- and Matrigel-iBMELCs. **a** FD4 permeability assay of LN221F- and Matrigel-iBMELCs on day 10. P_app_, apparent permeability coefficient. Data are presented as the mean ± SD (*n* = 6; **p < 0.01; Student’s *t*-test). **b** LY permeability assay. Data are presented as the mean ± SD (*n* = 6; **p < 0.01; Student’s *t*-test)

We attempted to identify basement membranes that were more suitable to promote the differentiation of iBMELCs than Matrigel using previously reported protocols (Fig. 1a). As shown in Fig. 1b, LN221F-iBMELCs had higher TEER values than the Matrigel-iBMELCs. In contrast, the TEER values of differentiated iBMELCs, which were cultured on Matrigel until day 8, were not influenced by coating LN221F from days 8 to 10 (Additional file 1: Figure S1A). To examine the effect of LN221F on human mature BMECs, we measured the TEER values of primary BMECs derived from human and monkey, and hCMEC/D3 (immortalized cell line) cells on several coating substrates including LN221F. The TEER value of human BMECs on LN221F was lower than those of human BMECs on LN511F (Additional file 1: Figure S1B). The TEER value of monkey BMECs on LN221F was lower than those of monkey BMECs on Matrigel, FBN, VTN-N, and LN511F (Additional file 1: Figure S1C). The TEER values of both human and monkey BMECs on LN511F were the highest among the six coating substrates. The TEER value of hCMEC/D3 cells on LN221F was almost equivalent to those of hCMEC/D3 cells on other matrices except Matrigel (Additional file 1: Figure S1D). We examined the TEER values of iBMELCs differentiated from human iPS cells at different seeding densities before start of differentiation because previous studies have shown that seeding density may affect barrier functions in iBMELCs [12]. The barrier function of LN221F was increased by a seeding cell density of < 60–70% confluency (the standard protocol) (Additional file 1: Figure S1E). Considering the possibility that coating substrates affected the state of undifferentiated iPS cells (days − 3 to 0), the gene expression levels of undifferentiation markers [octamer-binding transcription factor 4 (OCT-4) and NANOG] and cell number of iPS cells on day 0 were analyzed. These coating substrates hardly affected the state of human iPS cells, although LN221F and LN511F slightly increased NANOG mRNA expression in human iPS cells compared with Matrigel (Additional file 1: Figure S2A, B). The LN221F-iBMELCs derived from two iPS cell lines, 610B1 and 648A1, maintained high TEER values for a longer period than the Matrigel-iBMELCs (Fig. 1c). An important characteristic of BMECs is strict control of the permeability of substances via the paracellular route. Therefore, paracellular permeability of both LN221F- and Matrigel-iBMELCs was investigated. The results showed that the permeabilities of FD4 and LY were significantly lower in LN221F-iBMELCs derived from the two iPS cell lines as compared with that of the Matrigel-iBMELCs (Fig. 2a, b).

### LN221F-iBMELCs expressed BMEC-related markers

**Fig. 3:**
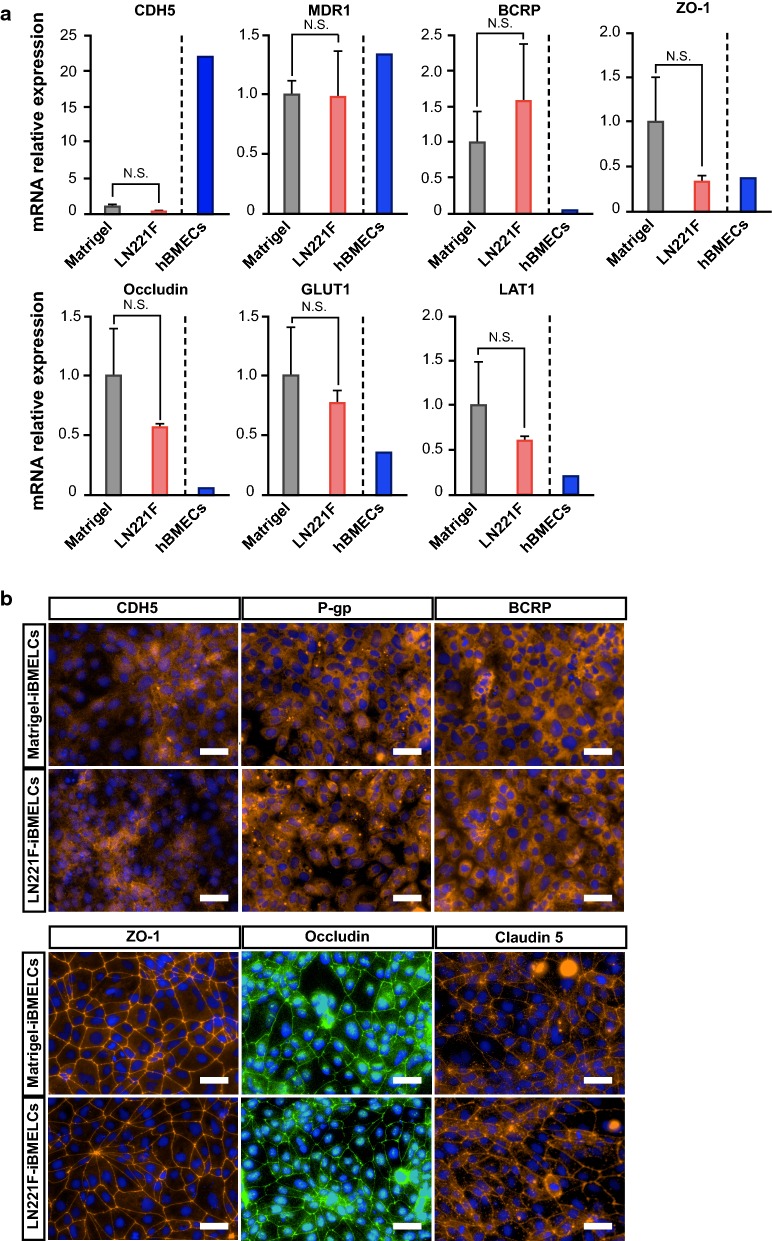
Characterization of LN221F- and Matrigel-iBMELCs. **a** Relative mRNA expression levels of CDH5, MDR1, BCRP, ZO-1, occludin, GLUT1, and LAT1 in LN221F- and Matrigel-iBMELCs derived from 610B1 on day 10. The values are normalized to those of HPRT1. The relative mRNA expression levels of Matrigel-iBMELCs were defined as 1. Data are presented as the mean ± SD (*n* = 3; N.S. = not significant; Student’s *t*-test). hBMECs: *n* = 1. **b** Immunofluorescence analysis of CDH5 (orange), P-gp (orange), BCRP (orange), ZO-1 (orange), occludin (green), and claudin 5 (orange) expression in LN221F- and Matrigel-iBMELCs derived from 610B1 on day 10. DAPI = blue. Scale bars = 50 μm

To assess whether LN221F-iBMELCs retain the characteristics of BMECs, gene expression levels in LN221F-iBMELCs, Matrigel-BMECs, and hBMECs were analyzed using RT-qPCR. As shown in Fig. 3a, the expression levels of CDH5 (a typical endothelial cell marker), MDR-1 (encoding P-gp) and BCRP (efflux transporters), ZO-1 and occludin (tight junction-related proteins), and glucose transporter 1 (GLUT1) and l-type amino acid transporter 1 (LAT1) (BMEC-related transporters) were similar in LN221F-iBMELCs and Matrigel-iBMELCs. However, CDH5 expression levels in both LN221F- and Matrigel-iBMELCs were lower than that in hBMECs. Other gene expression levels in LN221F- and Matrigel-iBMELCs were similar or greater as compared to hBMECs. Considering the low expression of CDH5, we additionally analyzed the expression levels of endothelial linage cell markers [platelet endothelial cell adhesion molecule (PECAM1), von Willebrand factor (vWF), and kinase insert domain receptor (KDR)] in both LN221F- and Matrigel-iBMELCs. The expression levels of these genes in both LN221F-iBMELCs and Matrigel-iBMELCs were lower than those in hBMECs (Additional file 1: Figure S3A). Besides, to assess whether both LN221F-iBMELCs and Matrigel-iBMELCs have the function of endothelial cells, we conducted Dil-Ac-LDL uptake assay. As a result, both LN221F-iBMELCs and Matrigel-iBMELCs incorporated Dil-Ac-LDL (Additional file 1: Figure S4A). To analyze the protein expression, we conducted immunofluorescence analysis. In visual observation, the protein localization and expression levels of CDH5, P-gp, BCRP, ZO-1, occludin, and claudin 5 in LN221F-iBMELCs were almost equivalent to those in Matrigel-iBMELCs (Fig. 3b). Furthermore, the cytosol fluorescence intensities of CDH5, P-gp, and BCRP immunostaining were almost equivalent to those in Matrigel-iBMELCs (Additional file 1: Figure S4B). Besides, Western blotting analysis confirmed that the protein expression levels of tight junction-related proteins (ZO-1, occludin, and claudin 5) in LN221F-iBMELCs were almost equivalent to those in Matrigel-iBMELCs (Additional file 1: Figure S4C). These results indicate that LN221F-iBMELCs exhibit characteristics of BMECs as well as Matrigel-iBMELCs. In addition, we analyzed the expression levels of mesodermal markers [T-box transcription factor T (TBXT) and KDR (both endothelial linage cell and mesodermal marker)] in the intermediate state of LN221F-iBMELCs and Matrigel-iBMELCs. The expression levels of these genes on day 3 in LN221F-iBMELCs were higher than those of Matrigel-iBMELCs. In contrast, the expression levels of these genes on day 6 in LN221F-iBMELCs were lower than those of Matrigel-iBMELCs (Additional file 1: Figure S3B). These results suggest that LN221F increases the rate of differentiation from iPS cells to iBMELCs.

### LN221F-iBMELCs have functions of efflux transporters

**Fig. 4:**
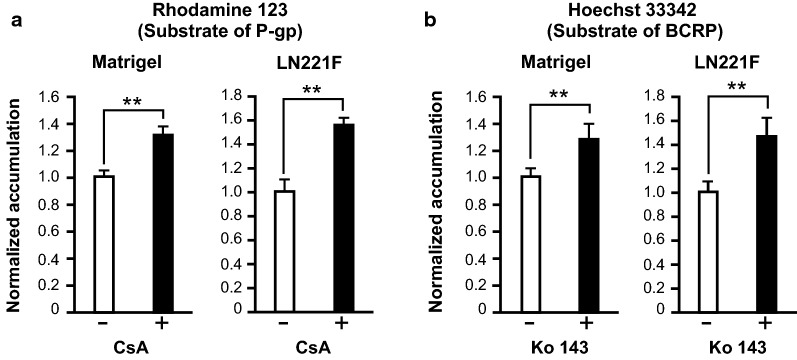
Analyses of P-gp and BCRP functions in LN221F- and Matrigel-iBMELCs. **a** Relative intracellular accumulation of rhodamine 123. The iBMELCs were incubated with 10 μM rhodamine 123 in the absence or presence of 10 μM CsA for 60 min at 37 °C. Relative fluorescence intensity values were normalized to the condition without inhibitor (set to 1). Data are presented as the mean ± SD (*n* = 6; **p < 0.01; Student’s *t*-test). **b** Relative intracellular accumulation of Hoechst 33342. The iBMELCs were incubated with 10 μM Hoechst 33342 in the absence or presence of 20 μM Ko 143 for 60 min at 37 °C. Relative fluorescence intensity values were normalized to the condition without inhibitor (set to 1). Data are presented as the mean ± SD (*n* = 6; **p < 0.01; Student’s *t*-test)

To determine whether LN221F-iBMELCs have functions of efflux transporters, which are essential for BBB functions, an intracellular accumulation assay of the substrates was conducted. The results showed that accumulation of rhodamine 123 (a P-gp substrate) and Hoechst 33342 (a BCRP substrate) in both Matrigel- and LN221F-iBMELCs was increased by the inhibitors CsA and Ko 143, respectively (Fig. 4a, b), which confirmed that both cell types had functions of P-gp and BCRP.

## Discussion

The results of the present study showed that fully defined coating matrix LN221F is more suitable for the differentiation of iBMELCs than Matrigel. The components of Matrigel can vary among lots and coating requires complex manipulation, whereas there is limited variation among lots of LN221F (imatrix 221). Besides, LN221F-iBMELCs maintained robust barrier function for a longer period than Matrigel-iBMELCs, whereas mRNA and protein expression levels of BMEC-related markers were similar to those of Matrigel-iBMELCs. Moreover, LN221F-iBMELCs as well as Matrigel-iBMELCs had functions of P-gp and BCRP. To enhance barrier integrity, iBMELCs are often co-cultured with BBB components, such as brain pericytes, astrocytes, and neurons [9, 13]. In this study, barrier integrity was successfully enhanced just by differentiation with the use of LN221F without a co-culture system. Based on these findings, we concluded that coating with LN221F is more suitable for iBMELC differentiation than Matrigel.

Laminin 221 is abundant in skeletal and heart muscles, as well as neuromuscular junctions [14, 15]. It has been reported that laminin 221 also induces differentiation of human embryonic stem cells to cardiovascular progenitors in vitro [15]. However, to our knowledge, few studies have investigated the relationship between laminin 221 and BBB development and maintenance. Therefore, before conducting the experiments, we speculated that basement membranes components that are abundant in BBB, such as FBN, LN411F, and LN511F [16], promote the differentiation of iBMELCs. However, unlike LN221F, these basement membranes components did not enhance the barrier function of iBMELCs as compared to Matrigel. The application of LN221F to mature iBMELCs from days 8 to 10 did not induce a tighter barrier. The TEER values of primary BMECs derived from human and monkey on LN221F were lower than those on some coating substrates. Of note, the TEER values of the primary cells on LN511F were the highest among the six coating substrates, suggesting that laminin 511 (or LN511F) was required for maintaining the integrity of the tight junction in mature BMECs. Besides, the TEER value of hCMEC/D3 cells on LN221F was higher than that on Matrigel but did not differ from those on other basement membranes such as FBN and LN511F. These findings suggest that LN221F is not always necessary as a basement membrane to enhance the barrier integrity of the mature BMECs and LN221F is particularly effective on the differentiation process of iBMELCs. There are two possible reasons why the contact between LN221F and human iPS cells during differentiation enhanced the barrier integrity of iBMELCs. First, the difference in the differentiation processes in vitro versus in vivo and iBMELCs require different factors for differentiation. Infact, iBMELCs expressed low levels of endothelial markers and have characteristics of both endothelial and epithelial cells [17]. In this study, iBMELCs were able to incorporate Dil-Ac-LDL, indicating that iBMELCs have the characteristics of endothelial cells. In contrast, CDH5, PECAM1, vWF, and KDR expression levels were lower in iBMELCs than hBMECs. Besides, the results of immunofluorescence staining showed that CDH5 was localized to the cytosol in iBMELCs, but is reportedly localized on the cellular membrane in various endothelial cell lineages, such as human umbilical vein endothelial cells [18], iPS cell-derived endothelial progenitor cells [19], human immortalized BMECs [20], and mouse BMECs [21]. Considering these findings, iBMELCs differentiated by a previously method or the modified method may not completely reflect the in vivo characteristics of endothelial cell lineages. As a second possibility, laminin 221 plays important roles in the development of the human BBB in vivo and influences the differentiation of iBMELCs in vitro. Indeed, laminin α2, which is a component of laminins 211 and 221, is indispensable for the development of the BBB as well as for maintaining barrier integrity [22]. Therefore, it is quite possible that laminin 221 has a positive effect on the differentiation and barrier function of iBMELCs in a laminin α2-dependent manner.

The results of the present study also confirmed that LN221F-iBMELCs have characteristics of BMECs, such as expression of BMEC-related markers and the functions of efflux transporters. However, the expression levels of genes and proteins related to tight junctions in LN221F-BMECs were equivalent to those of Matrigel-iBMELCs, even though LN221F-iBMELCs exhibited more robust barrier integrity than Matrigel-iBMELCs. The reasons for these findings will be elucidated in future studies.

## Conclusion

In this study, we succeeded in identifying basement membrane LN221F suitable for the differentiation of iBMELCs. LN221F-iBMELCs had a more robust barrier function for a longer period than that of Matrigel-iBMELCs. Furthermore, the gene and protein expression levels of BMEC-related markers in LN221F-iBMELCs were comparable to those of Matrigel-iBMELCs. Like Matrigel-iBMELCs, LN221F-iBMELCs also had functions of P-gp and BCRP, which are important efflux transporters of the BBB. In addition, the components of Matrigel can vary among various batches, whereas limited variation occurs among the batches of LN221F. Therefore, we consider that coating with LN221F, which requires simple manipulation, as an alternative to Matrigel is useful for the reproducible production of large numbers of iBMELCs for pharmacokinetic and pathological models of the BBB.

## Supplementary information

**Additional file 1.** Additional figures.

## Data Availability

The datasets used and/or analyzed in the current study are available from the corresponding author upon reasonable request.

## Abbreviations

BCRP: Breast cancer resistance protein
BSA: Bovine serum albumin
CDH5: Cadherin 5
CsA: Cyclosporine A
Dil-Ac-LDL: 1,1′-Dioctadecyl-3,3,3′,3′-tetramethyl-indocarbocyanine perchlorate acetylated low-density lipoprotein
DMEM/F12: 1:1 mixture of Dulbecco’s modified Eagle’s medium and Ham’s nutrient mixture F-12
D-PBS: D-phosphate-buffered saline
FD4: Fluorescein isothiocyanate-dextran 4 kDa
FGF2: Fibroblast growth factor 2
GLUT1: Glucose transporter 1
HBSS: Hank’s Balanced Salt Solution
HEPES: 4-(2-Hydroxyethyl)-1-piperazineethanesulfonic acid
HE-SFM: Human endothelial-SFM
HPRT1: Hypoxanthine guanine phosphoribosyltransferase 1
iPS cells: Induced pluripotent stem cells
KDR: Kinase insert domain receptor
KSR: KnockOut Serum Replacement
LAT1: l-type amino acid transporter 1
LN221F-iBMELCs: Human induced pluripotent stem cell-derived brain microvascular endothelial-like cells differentiated on laminin 221 fragment
LA221F: Laminin 221 fragment
LN411F: Laminin 411 fragment
LN511F: Laminin 511 fragment
LY: Lucifer yellow
Matrigel-iBMELCs: Human induced pluripotent stem cell-derived brain microvascular endothelial-like cells differentiated on Matrigel
MDR1: Multiple drug resistance 1
PECAM1: Platelet endothelial cell adhesion molecule
P-gp: P-glycoprotein
RA: All-*trans* retinoic acid
SFM: Serum-free medium
TBXT: T-box transcription factor T
TEER: Transendothelial electrical resistance
vWF: Von Willebrand factor
ZO-1: Zonula occludens-1
