# Supplementary material for: Laminin 221 fragment is suitable for the differentiation of human induced pluripotent stem cells into brain microvascular endothelial-like cells with robust barrier integrity
Source: Fluids Barriers CNS. 2020 Mar 30;17:25. doi: 10.1186/s12987-020-00186-4 (PMC7106710; doi:10.1186/s12987-020-00186-4)
Supplement: Supplementary file 1 — Additional file 1. Additional figures. [file 12987_2020_186_MOESM1_ESM.pdf]

# Additional file 1

# Laminin 221 fragment is suitable for the differentiation of human induced pluripotent stem cells into brain microvascular endothelial-like cells with robust barrier integrity

Hiromasa Aoki, Misaki Yamashita, Tadahiro Hashita, Takahiro Iwao, Tamihide Matsunaga

**Components**
Four Supplemental Figures

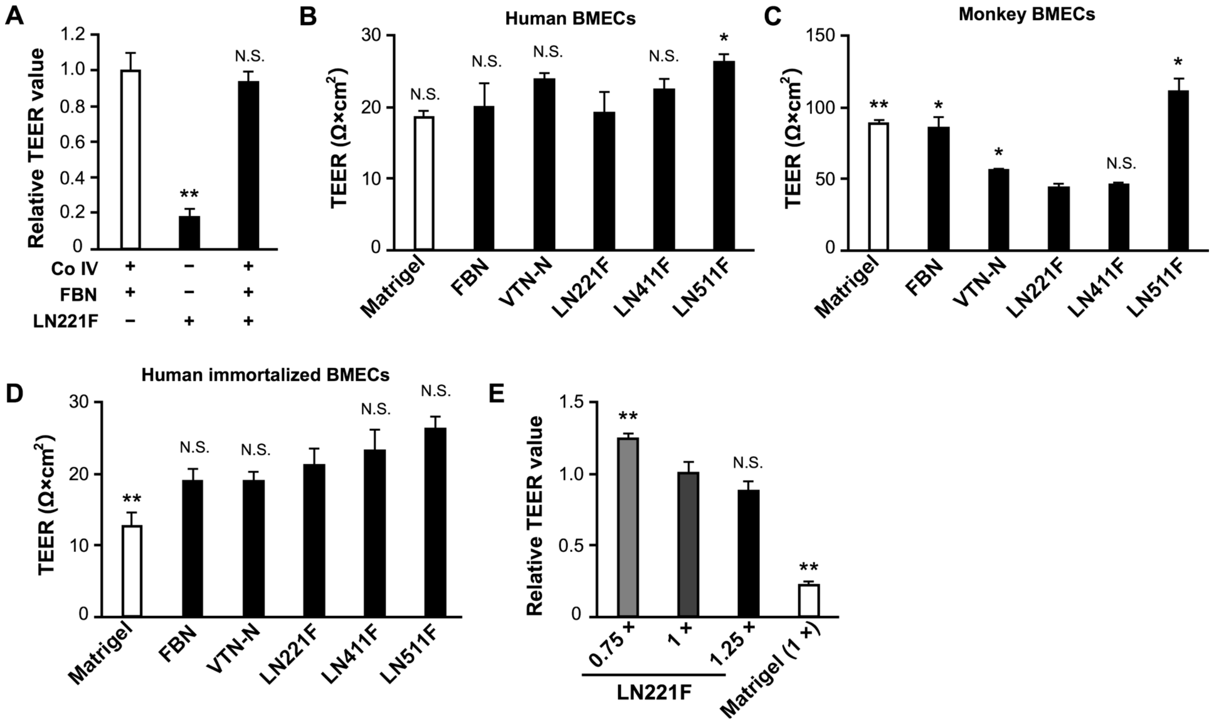


**Fig. S1. Analyses of the effect of LN221F under various conditions**

(A) Measurement of TEER values of 610B1-derived Matrigel-iBMELCs seeded on inserts coated with LN221F alone, a mixture of collagen type IV and FBN, or a mixture of collagen type IV, FBN, and LN221F (days 8–10). The relative TEER value of 610B1-derived Matrigel-iBMELCs seeded on inserts coated with a mixture of collagen type IV and FBN was defined as 1. Data are presented as mean ± SD ($n = 3$; N.S. = not significant, $^{**}p < 0.01$; Tukey's Honest Significant Difference test, collagen type IV, and fibronectin-coated insert group vs. others).

(B) Measurement of TEER values of BMECs derived from human (day 4) on Matrigel, FBN, VTN-N, LN221F, LN411F, or LN511F. Data are presented as mean ± SD ($n = 3$; N.S. = not significant, $^{*}p < 0.05$; Tukey's Honest Significant Difference test, LN221F group vs. others).

(C) Measurement of TEER values of BMECs derived from *Macaca irus* (day 4) on Matrigel, FBN, VTN-N, LN221F, LN411F, or LN511F. Data are presented as mean ± SD ($n = 3$; N.S. = not significant, $^{*}p < 0.05$, $^{**}p < 0.01$; Games–Howell test, LN221F group vs. others).

(D) Measurement of TEER values of hCMEC/D3 cells (day 4) on Matrigel, FBN, VTN-N, LN221F, LN411F, or LN511F. Data are presented as mean ± SD ($n = 3$; N.S. = not significant, $^{**}p < 0.01$; Tukey's Honest Significant Difference test, LN221F group vs. others).

(E) The TEER values of 610B1-derived LN221F-iBMELCs on day 10 depending on seeding cell density before the start of differentiation. The seeding cell number were set to $6 \times 10^5$ cells/well (0.75 × group), $8 \times 10^5$ cells/well (1 × group), or $10 \times 10^5$ cells/well (1.25 × group). The relative TEER value of 1 × group was defined as 1. Data are presented as mean ± SD ($n = 3$; N.S. = not significant, $^{**}p < 0.01$; Tukey's Honest Significant Difference test, 1 × group vs. others).

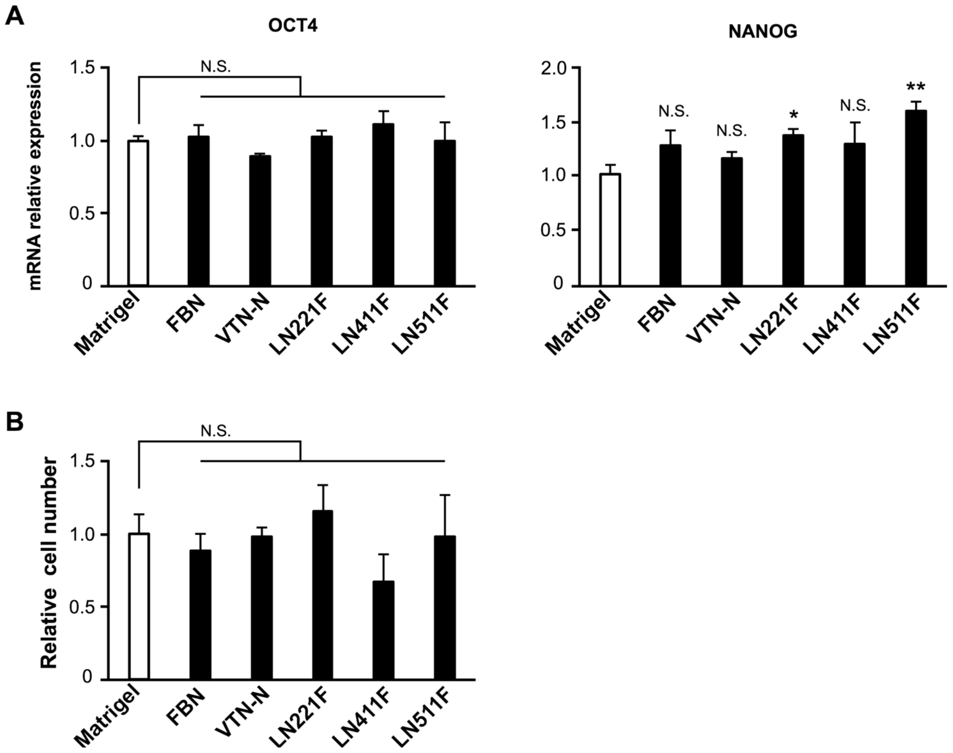


**Fig. S2. Analyses of the effects of Matrigel, FBN, VTN-N, LN221F, LN411F, or LN511F on human iPS cells**

(A) Relative mRNA expression levels of OCT-4 and NANOG in human iPS cells cultured on Matrigel, FBN, VTN-N, LN221F, LN411F, or LN511F from days −3 to 0. The values are normalized to those of HPRT1. The relative mRNA expression levels of 610B1 cultured on Matrigel were defined as 1. Data are presented as mean ± SD ($n$ = 3; N.S. = not significant, *$p < 0.05$, **$p < 0.01$; Tukey's Honest Significant Difference test or Games–Howell test, Matrigel group vs. others).

(B) Relative cell viabilities (610B1) on day 0 cultured on Matrigel, FBN, VTN-N, LN221F, LN411F, or LN511F from days −3 to 0 were analyzed by CCK-8 assay. The relative cell number of 610B1 cultured on Matrigel was defined as 1. Data are presented as mean ± SD ($n$ = 3; N.S. = not significant; Games–Howell test, Matrigel group vs. others).

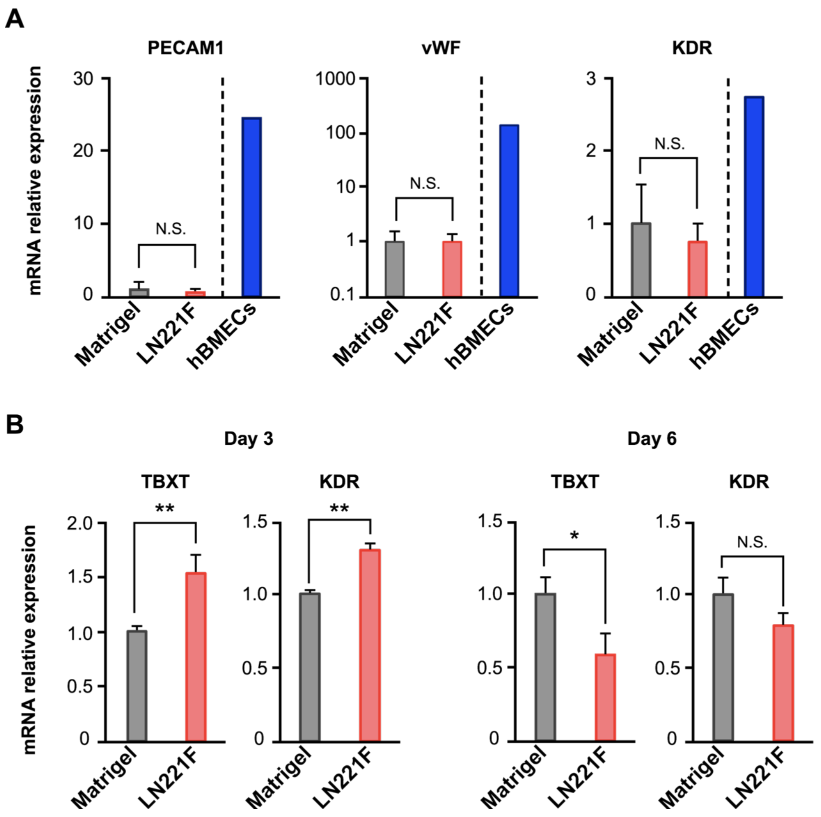


**Fig. S3. Gene expression analyses of endothelial cell markers in iBMELCs and mesodermal markers in the intermediate state of iBMELCs**

(A) Relative mRNA expression levels of PECAM1, vWF, and KDR in LN221F-iBMELCs and Matrigel-iBMELCs from 610B1 on day 10. The values are normalized to those of HPRT1. The relative mRNA expression levels of Matrigel-iBMELCs were defined as 1. Data are presented as mean ± SD ($n = 3$; N.S. = not significant; Student's *t*-test).

(B) Relative mRNA expression levels of TBXT and KDR in differentiated cells (on day 3 or 6) derived from 610B1. The values are normalized to those of HPRT1. The relative mRNA expression levels of Matrigel-iBMELCs were defined as 1. Data are presented as mean ± SD ($n = 3$; N.S. = not significant, $*p < 0.05$, $**p < 0.01$; Student's *t*-test).

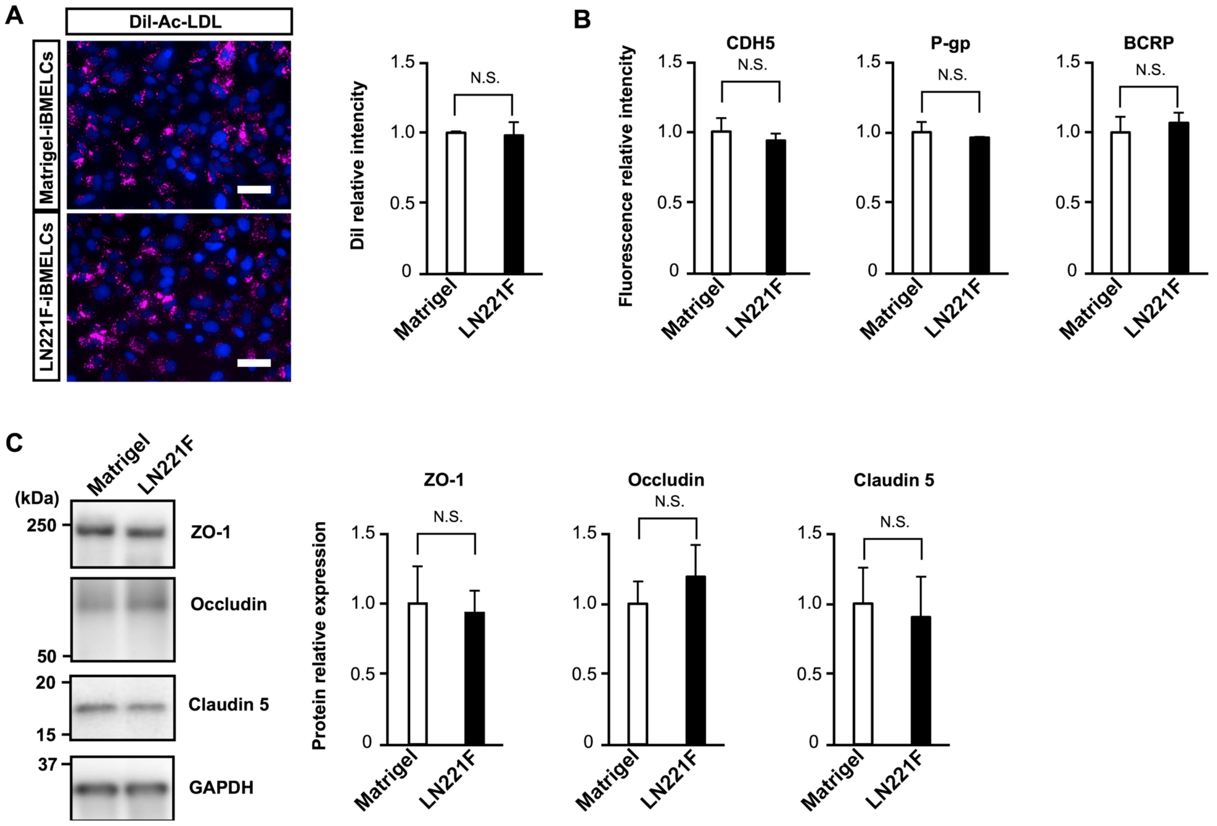


**Fig. S4. Quantification of protein expression and uptake of Dil-Ac-LDL**

(A) Uptake of Dil-Ac-LDL by iBMELCs on day 10 derived from 610B1. Dil-Ac-LDL = pink; Hoechst 33342 = blue. Scale bars = 50 µm. Mean fluorescence intensities of Dil-Ac-LDL on cellular cytosol. The mean fluorescence intensity of Matrigel-iBMELCs was defined as 1. Data are presented as mean ± SD ($n$ = 3, 6 fields/well).

(B) Mean fluorescence intensities of CDH5, P-gp, and BCRP immunostaining of cellular cytosol. The mean fluorescence intensities of Matrigel-iBMELCs were defined as 1. Data are presented as mean ± SD ($n$ = 3, 6 fields/well; N.S. = not significant; Student's $t$-test).

(C) The protein expression levels of ZO-1, occludin, and claudin 5 in Matrigel- and LN221F-iBMELCs on day 10 derived from 610B1 were subjected to western blot analysis. The protein expression levels were normalized to GAPDH levels. The protein expression levels of Matrigel-iBMELCs were defined as 1. Data are presented as mean ± SD. ($n$ = 3; N.S. = not significant; Student's $t$-test).
